# Predicting resprouting of Platanus × hispanica following branch pruning by means of machine learning

**DOI:** 10.3389/fpls.2024.1297390

**Published:** 2024-03-07

**Authors:** Qiguan Shu, Hadi Yazdi, Thomas Rötzer, Ferdinand Ludwig

**Affiliations:** ^1^ Professorship for Green Technologies in Landscape Architecture, TUM School of Engineering and Design, Technical University of Munich, Munich, Germany; ^2^ Chair for Forest Growth and Yield Science, Technical University of Munich, Freising, Germany

**Keywords:** tree manipulation, branch pruning, resprout pattern, TLS, tree QSM, machine learning

## Abstract

**Introduction:**

Resprouting is a crucial survival strategy following the loss of branches, being it by natural events or artificially by pruning. The resprouting prediction on a physiological basis is a highly complex approach. However, trained gardeners try to predict a tree’s resprouting after pruning purely based on their empirical knowledge. In this study, we explore how far such predictions can also be made by machine learning.

**Methods:**

Table-topped annually pruned Platanus × hispanica trees at a nursery were LiDAR-scanned for two consecutive years. Topological structures for these trees were abstracted by cylinder fitting. Then, new shoots and trimmed branches were labelled on corresponding cylinders. Binary and multiclass classification models were tested for predicting the location and number of new sprouts.

**Results:**

The accuracy for predicting whether having or not new shoots on each cylinder reaches 90.8% with the LGBMClassifier, the balanced accuracy is 80.3%. The accuracy for predicting the exact numbers of new shoots with the GaussianNB model is 82.1%, but its balanced accuracy is reduced to 42.9%.

**Discussion:**

The results were validated with a separate dataset, proving the feasibility of resprouting prediction after pruning using this approach. Different tree species, tree forms, and other variables should be addressed in further research.

## 1 Introduction

Disturbances to tree growth, like ice storms, fires, wind, and diseases, are common in nature (Hauer et al., 2006; Simler et al., 2018). They cause great loss in trees’ biomass, especially above the ground. In view of this, resprouting is a vital survival strategy for most tree species: new shoots can grow out of dormant buds rapidly at certain positions after the disturbance. This process is recognized as a major force in forest regeneration (Matula et al., 2019) and significantly impacts forest dynamics (Martini et al., 2008). Humans recognized and harnessed these phenomena from early times (Petit and Watkins, 2003; Candel-Pérez et al., 2022). A famous example is pollarding, where all the shoots of a tree crown are regularly cut off to encourage the growth of new sprouts, which were used as firewood and material for weaving baskets.

Regardless of the practical use, it is a highly interesting but, at the same time, a very complex challenge to understand and predict the resprouting patterns of trees caused by disturbances on a physiological basis. These patterns are firstly determined by axillary buds, which either form new shoots or enter dormancy (Suzuki, 2002). This “decision” is essentially controlled by hormone signals. Auxin was considered one of the primary mediators in the 20th century, while new findings indicate that cytokinins (Salam et al., 2021; Schneider et al., 2022) and strigolactones (Gomez-Roldan et al., 2008) play a major role in apical dominance and branching inhabitation respectively. Without a clear conclusion yet regarding their exact mechanisms, studies tried to understand resprouting patterns from other micro and macro perspectives: its relation to genetic regulation (Hill and Hollender, 2019), in responding to seasonal adaptation (Singh et al., 2022), or by an explanation known as Low Energy Syndrome (Martín-Fontecha et al., 2018).

However, these endogenous physiological processes do not tell the whole story of resprouting. Leaf area and light are redistributed after the disturbances, which then affects photosynthetic processes (Balandier et al., 2000). This does not simply mean a decrease in photosynthetic capacities but involves the reallocation of carbon- and other resources among plant organs such as fruits (Kohek et al., 2015; Tosto et al., 2023) and flowers (Grechi et al., 2022). What makes the impact of this disturbance even more complex is timing. For example, summer pruning on an apple tree typically causes a temporary loss of apical dominance and an increase in its cytokinin supply. But depending on its exact timing, the dominance may be delayed or even prevented (Saure, 1987). As a result, a precise analysis of how a disturbance reshapes a tree using a physiological approach must address the primary status of the hormone, resource reallocation, and the timing issue. To our knowledge, no research has brought all these aspects together so far.

Even without any precise analytical tools regarding resprouting analysis, skilled practitioners learn how to prune a tree in their charge. They neither measure its sap-flows with multiple sensors nor meter the cytokinin concentration in chemistry labs. By going around the tree and observing the main branches, they decide where to prune. Their decisions are based on empirical knowledge of natural phenomena, derived initially from accurate observations of causes and effects – the tree’s resprouting reaction to the loss of branches by pruning. Countless repetitions of similar processes have been experimented in horticulture over centuries (Saunders, 1898). For a gardener, their primary pruning skills may start with a set of general rules written in a manual book (Brickell and Joyce, 1996). Then, their skills will independently evolve further through repeated work practices specific to different climate zones, species, etc. Suppose their pruning decisions lead to resprouting reactions largely similar to their expectations, gardeners finally prove to be able to predict the tree’s response purely on visual observation and geometrical patterns without digging deep into simulating physiological processes.

In horticulture and arboriculture, we currently see a strong trend toward the automation of pruning by machines or robots (Sam et al., 2022). So far, these are comparatively standardized actions (Li et al., 2021; Sam et al., 2022), but the more complex the tasks become in this regard, the more important is a plausible, robust, and prompt prediction of the growth reaction of a tree to pruning. At the same time, it can be assumed that in the future, trees worldwide will increasingly experience growth disturbances due to the consequences of climate change (drought, stronger and more frequent storms), which will be coupled with a loss of biomass and subsequent resprouting. In order to assess the development of such trees, for example, in an urban context, also here a plausible, robust, and prompt prediction of resprouting in response to the previous loss of branches and twigs is necessary.

In this regard, physiological forecasts seem to be too complex, rely on too many often-unknown parameters (e.g., weather), and thus are likely to be too sensitive to errors and too slow [in reference to, i.e., the applications in forecasting building energy performance (Chakraborty and Elzarka, 2019; Fathi et al., 2020)]. The study at hand aims to develop the basics for a prediction model on the basis of geometric patterns corresponding to the approach of experienced gardeners using a concrete example.

Rapid development in remote sensing is providing a solid base for this aim. First of all, terrestrial LiDAR scanners can capture detailed geometry of objects with a precision of up to 3 mm from multiple standing positions (RIEGL, 2023). This method proves capable of capturing a tree’s trunk and branches with more than 10 mm diameter (Gobeawan et al., 2018; Yang et al., 2022) during its leaf-off state (Kükenbrink et al., 2022). Raw data is stored in the form of a discrete point cloud. Furthermore, different approaches have been developed to extract tree structure: skeleton abstraction following occupancy grids (Bucksch et al., 2010; Sun et al., 2022); branch direction by eigenvectors of point patches or sections (Bremer et al., 2013; Raumonen et al., 2013); skeleton as the Dijkstra’s shortest path from the tree base to ends (Du et al., 2019; Li et al., 2022, 2022; Wang et al., 2014); skeleton redrawn with searching steps (Hackenberg et al., 2014); learning the reconstruction pattern through a neural network (Liu et al., 2021). Overall, this abstracted information about tree architecture is called the quantitative structure model (QSM) (Åkerblom et al., 2017; Shu et al., 2022). In this way, every segment of the tree stem or branch can be retrieved, containing its diameter, length, axial direction, and hierarchy in the whole branching structure, as well as the pointer to its parent and child segments.

These data for a computational model can be compared with human experiences. The process for an algorithm to “learn from experience” without being explicitly programmed was defined as machine learning (Samuel, 1959). Over 70 years of development, machine learning models have proven capable and efficient to inherently solve the 5 typical problems of data science, namely classification, anomaly detection, regression, clustering, and reinforcement learning (Alzubi et al., 2018). Among them, classification models assign class labels to testing instances where the high dimensional predictor features are known (Kotsiantis, 2007). Specific to our research, QSMs provide the high dimensional features for describing tree segments while the resprouting response of the trees are the class labels. In handling them, the classification models have the advantage of 1) capturing intricate and non-linear patterns within data autonomously (i.e., Hassona et al., 2021). The resprouting patterns are likely to be non-linear to features in QSMs (see section 2.4). 2) They work for both binary and multi-class classification problems (i.e., Teimoorinia et al., 2020). The position of new shoots is a binary problem, while the number of new shoots is a multi-class problem. 3) They have good scalability to large datasets (Gupta et al., 2016). The total number of tree segments can be large (see section 2.2). 4) They can self-update through new training datasets. This allows the prediction to improve its accuracy or be adapted to more species and forms if having corresponding data (see section 4). Additionally, from a practical aspect, open-source packages such as scikit-learn (Pedregosa et al., 2011) have integrated common classification models of machine learning, offering easy access to adapt parameters for different applications. These characteristics collectively make machine learning an attractive and powerful approach for addressing resprouting prediction of trees following pruning.

Equipped with the digital tools above, accurate information regarding tree structures can be collected and processed in analogy to what a real gardener does. Building on this, we are addressing the following questions: How can we predict the position and number of resprouting shoots based on a purely “visual approach” (pattern recognition)? Which machine learning model achieves the best accuracy for this task?

## 2 Materials and methods

### 2.1 Study case

**Figure 1:**
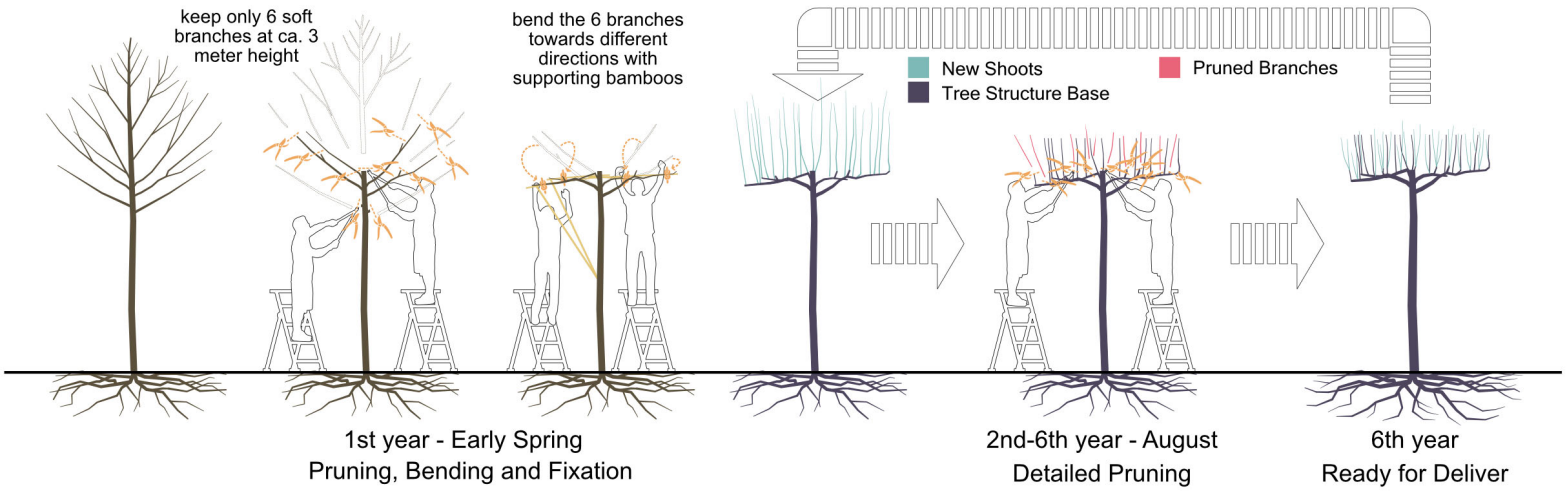
The procedure for producing a table-topped *platanus* through iterative branch and shoot selection and pruning with an intensive labour force.

To address our questions, we looked for tree cases that are frequently pruned in a distinct manner under similar environmental conditions. At Bruns Nursery, Bad Zwischenahn in north Germany, so-called table-topped plane trees (*Platanus × hispanica*) are grown in a clearly defined area under standardized conditions. The crowns of these trees are shaped into a flat layer through labour-intensive maintenance. This form probably originates from Baroque gardens, where plants were kept in an orthogonal manner to enhance the orientation or perspective (Dobrilovič, 2010). Due to the expansion of the crown like an umbrella, it is still used in European cities nowadays for shading squares and pedestrian areas (e.g., the central square at Labouheyre, France). To produce such trees, there are two phases in general. In the first phase, a young plane tree with a naturally grown canopy is intensively trimmed. At around 3 meters in height, six branches are selected and bent horizontally into different directions with equal angles in between. Where necessary, bamboo sticks are added as temporary supports to force the branch into the aimed direction (see “1^st^ year” in **Figure 1**). In the second phase, new shoots or even some of the older shoots from these six main branches are carefully selected and pruned by experienced gardeners. Pruning decisions are important at this phase to enable shoot growth only at desired positions. Some shoots reserved from previous years could still be trimmed off if there appears another new shoot that becomes a better option. This procedure is repeated in the following years (see “2^nd^-6^th^ year” in **Figure 1**). Multiple reiterations of the tree by resprouting result in a complex branching pattern. Due to the annual pruning and relatively complex branching pattern, the second phase of these cases is considered effective in analysing the abilities of machine learning models in predicting resprouting patterns based on quantitative structural tree models under complex yet repetitive conditions. It should be noticed that the aim of this study is not recreating this specific form of tree geometry like the table-topped *Platanus × hispanica* but to gain fundamental knowledge regarding resprouting reactions of trees.

### 2.2 Data acquisition and pre-processing

**Figure 2:**
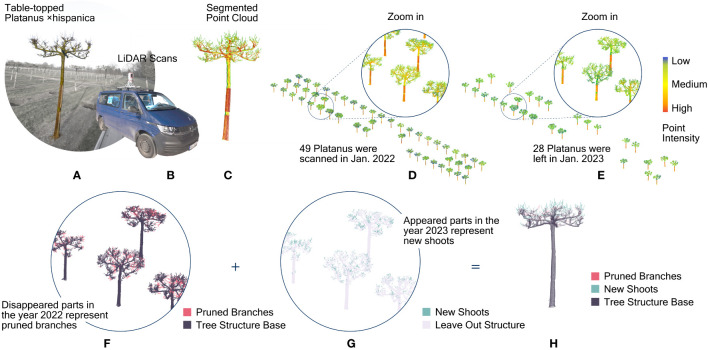
The overall procedure for detecting pruned branches and new shoots from point clouds of LiDAR scans in two consecutive years. **(A)** A photo of the table-topped plane trees grown at the nursery; **(B)** LiDAR scanner mounted on the vehicle; **(C)** segmented point cloud of the tree shown in the photo; **(D)** the segmented point clouds of individual plane trees in 2022; **(E)** the segmented point clouds of individual plane trees in 2023; **(F)** labeled points representing the pruned branches on the point clouds acquired in 2022; **(G)** labeled points representing the new shoots on the point clouds acquired in 2023; **(H)** an integrated point cloud with points labeled as unchanged structure base, pruned branches and new shoots.

In the subsequent two winters, namely in January 2022 and January 2023, an area consisting of 3- and 4-year-old table-topped Platanus (see **Figure 2A**) planted in 3 rows at Bruns Nursery were scanned with LiDAR scanner RIEGL VZ-400i. The scanner was mounted on a tripod in 2022, while mounted on a vehicle (see **Figure 2B**) in 2023. All the scans were set to the “Panorama30” standard (with angular resolution 0.030°) and conducted in a “stop-and-go” method. Scanning positions were located along each row at every third tree (ca. 12 m). Point clouds from different scan positions were automatically registered in RiSCAN Pro in reference to GNSS coordinates recorded with Leica Zeno FLX100 plus (Leica, 2023; Yazdi et al., 2024). The original GNSS coordinates indicate accuracies ranging between 0.68 to 0.80 m at different scan positions. Therefore, the reliability of GNSS was set to low during the automatic registration and the multistation adjustment. With all the steps above, we got two point clouds containing all the tree cases for the years 2022 and 2023, respectively. Afterward, individual trees were segmented manually (see **Figure 2C**). This manual step is efficient for our cases because those trees planted in the nursery were almost perfectly aligned at an equal distance, and their crowns did not touch each other. The ground surface was flat and clear. There were no irrelevant objects, such as shrubs around tree trunks. A total of 49 plane trees were scanned in 2022 while the number of trees scanned in 2023 was 28 (due to tree sales during 2022, see **Figures 2D, E**). As a result, we got point clouds of 28 plane trees for both years.

The next step was to identify changes in the geometrical structure of the trees in these two years (see **Figure 2H**). For this purpose, the two corresponding scans regarding the same trees must be aligned. The GNSS coordinates have an offset of up to 0.8 meters, which is insufficient for our demand. The most common algorithm for matching 3d models precisely, namely Iterative Closest Point (Rusinkiewicz and Levoy, 2001), does not work for these tree cases because the new shoots and the extensive pruning on tree branches have altered their geometries significantly. A supervised alignment by manually picking point pairs on corresponding branch surfaces also caused visible deviations owing to the girth growth. Finally, we manually aligned all tree pairs individually using multiple views. This guaranteed the best possible alignment despite significant geometrical changes between the two scans. Only then were we able to precisely detect the changes caused by growth and pruning between the point clouds. In principle, point sets that only appeared in the scan of 2022 and disappeared in the scan of 2023 should represent branches pruned away. Conversely, point sets that were only found in 2023 should represent new shoots. In practice, an object has no identical points on its surface in two independent scans. To identify geometrical changes on the two point clouds, cloud-to-cloud distance (Jafari et al., 2017) was applied. For each point in one point cloud, this function calculates its distance to its nearest neighbour in the other point cloud using the Hausdorff distance (Taha and Hanbury, 2015). This calculation was conducted in CloudCompare, where the octree level is set to “auto” (Girardeau-Montaut, 2023). Based on the cloud-to-cloud distance values, a minimum distance threshold ranging between 0.020 to 0.045 m was customized to each point cloud for segmenting unchanged and changed tree segments (see **Figures 2F, G**). When the alignment of the tree was precise, and little noise was around the branches, the threshold was set smaller to tell apart more accurate changes. Points whose distances were larger than the thresholds represent tree segments that do not exist in the other scan. For those points in the scan of 2022, those changed points represent pruned branches, while those points in the scan of 2023 represent new shoots (see **Figure 2H**).

**Figure 3:**
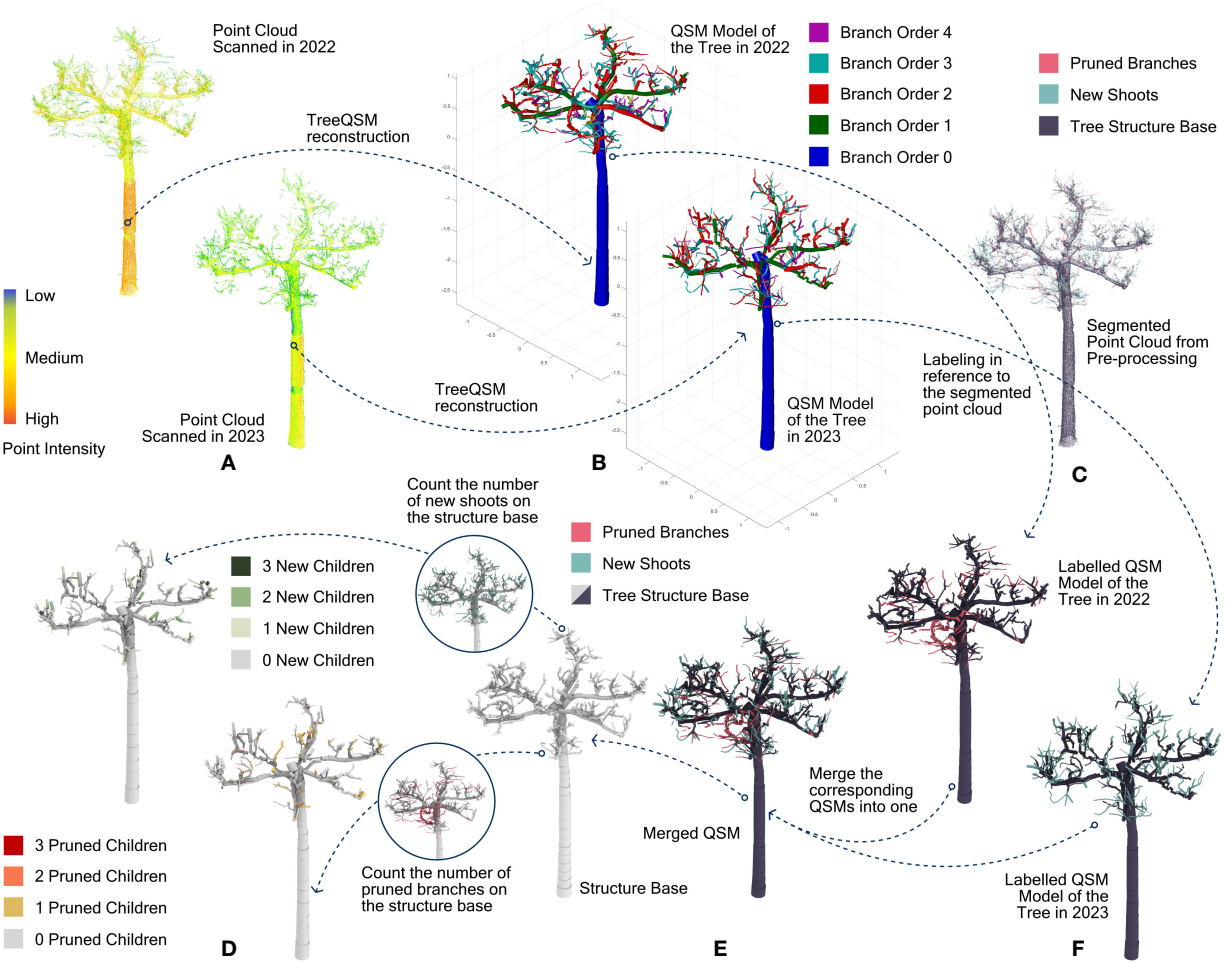
The overall procedure for labelling and reorganizing the dataset. **(A)** point clouds of the same tree scanned in 2022 and 2023 respectively; **(B)** QSM models out of the point clouds; **(C)** labeled point cloud as the reference; **(D)** final labeled QSM dataset consisting of the unchanged structure base and the numbers of their pruned and new children cylinders; **(E)** integrated QSM with cylinders labeled as unchanged structure base, pruned branches and new shoots. **(F)** labeled QSMs representing the trees scanned in 2022 and 2023, respectively.

Parallel to change detection, the point clouds were also used to create quantitative structural models (QSMs) of the trees (see **Figure 3A**) by TreeQSM (Raumonen et al., 2013) in MATLAB (The MathWorks Inc., 2023) (see **Figure 3B**). Raumonen et al. (2013) integrated multiple automatic steps in this pipeline to recreate precise cylindrical models out of the dense point cloud of an individual tree. The main steps are defining small sets of patches on tree surfaces; segmenting patches into a trunk and branches using iterative searching steps; fitting cylinders on point clouds of the same branch; optimizations to reduce the error caused by noises and occultations; generating statistics on cylinders and the tree. Besides TreeQSM, some other open-source QSM reconstructing tools like AdTree (Du et al., 2019) and AdQSM (Fan et al., 2020) build tree structures by the Dijkstra’s shortest path and the minimum spanning tree, respectively. In primary tests by the authors, they appeared to be more sensitive to outliers in our dataset. Primarily when they built detailed twigs at the branch’s high end, shoots were invented on fake skeletons initiated by the outliers in the point clouds. Therefore, they did not reflect the actual sprouting pattern. Compared to them, TreeQSM fits only cylinders to point patches in defined sizes. This approach performs better in noise and outlier resistance than those methods using Dijkstra’s shortest path and the minimum spanning tree, being the most faithful in describing the accurate tree geometries among the mentioned tools. One limitation of the TreeQSM tool lies in the robustness of the branch segmentation due to some random seeds in patch generation. Following the manual book (Raumonen, 2022), we tested 18 configurations of different settings regarding the patch sizes for reconstructing the QSMs in TreeQSM on each point cloud. For each configuration further, the reconstruction was repeated 15 times to reduce the impacts of pseudo-random numbers. Finally, the QSM with minimum mean distances from points to trunk and branch cylinders was chosen as the model for the corresponding point cloud using the embedded function named “select_optimum”. It should be addressed again that in our dataset, each tree is represented with two different point clouds and two QSMs accordingly, showing their stands in 2022 and 2023 respectively. To further ensure a precise reconstruction, the outliers were pre-deleted through the statistical outlier removal (SOR) tool (Rusu and Cousins, 2011). This step was implemented in CloudCompare, where the number of points used for mean distance estimation was set to 6. The standard deviation multiplier threshold was set to 1.

### 2.3 Labelling and reorganizing the dataset

In the pre-processing, the trimmed branches and the new shoots were detected in the point clouds, while topological cylinders were generated with TreeQSM. The next step was to combine these two datasets. The individual cylinders of the QSMs must be labelled as to whether they are part of an unchanged branch (not considering the girth growth), a pruned branch or a new shoot. This was achieved by using a distance threshold between points of the cylindrical axis and their nearest neighbouring point of the segmented point clouds. For our data, we examined only every cylinder’s start and end point. If the sum of their mean distances to their 10 nearest neighbours with the same label (i.e., trimmed branches) was below 100 mm, this cylinder was labelled the same (see **Figures 3C, F**). To enhance the accuracy of the labelling, three more criteria were added based on practical rules when pruning these trees: for any cylinder labelled as part of either a new shoot or a pruned branch, its radius must be smaller than 20 mm (one-year-old shoots do not reach more than 20 mm in diameter for the trees at hand); for any cylinder labelled as part of a pruned branch, its branch hierarchical order must be larger than 1 (not the tree trunk and the primary branch); the label for trimmed branches and new shoots on one cylinder is passed on to all its children cylinders.

After labelling, the cylinders of different labels (unchanged branches, pruned branches and new shoots) are still separated in two QSMs regarding the same tree. There is no correspondence between these two QSMs, as their reconstruction processes are independent. Therefore, cylinders of the trimmed branches in one QSM must be integrated into the other QSM that contains the main tree structure and the new shoots, or reversely, cylinders of new shoots must be integrated into the QSM with the trimmed branches. This is a tricky process. While the geometric data remain the same for every cylinder, its topological data regarding the ID of the cylinder, its parent cylinder, and its child cylinder must be corrected, as well as the branch order and its position in the branch. Regarding whether to transfer cylinders of new shoots or pruned branches to the other QSM, considerations can be described as follows. The pruned branches, in general, could only be the same size or thicker than the new shoots. Consequently, cylinders of pruned branches have higher robustness in their position through cylinder fitting. As a result, the certainty of redefining their topological parent in another QSM based on their relative positions is supposed to be higher. So, for our dataset, the cylinders of pruned branches were picked out from their original QSM and integrated into the other QSM that has the new shoot cylinders (see **Figure 3E**). Their new parent cylinders were redefined as those whose endpoints were located closest to their starting point. Based on this, the topological data for every single cylinder in the newly merged QSM were entirely overwritten due to this change.

Finally, the total number of pruned branches and new shoots on every cylinder was counted (see **Figure 3D**). This became the crucial attribute for the prediction models in the next step.

### 2.4 Prediction with various classification models

After all the processes described above, the dataset contains 34,245 items, representing 28 table-topped plane trees. Each item corresponds to one cylinder, which contains the following attributes: tree’s ID; cylinder’s ID; parent cylinder’s ID; child cylinder’s ID in the same branch; x-y-z coordinate of the cylinder start; a normalized 3d vector of the axial direction; branch’s ID; its sequence in the branch; branch order; cylinder length; cylinder radius; the number of pruned children and new children; the Boolean value if this cylinder is virtually added during QSM reconstruction; the Boolean value if this cylinder is pruned out.

The relationships between each two attributes (except for the IDs and Boolean values) are illustrated in **Supplementary Figure 2**. For our research purpose, the sprout location and numbers are the labels of new shoots on each cylinder. We tested classification models in machine learning to find links between these topological and geometrical attributes and the predicting target. Among these target labels, 16,183 (47.3%) cylinders were labelled “-1”, meaning that they were trimmed away. These cylinders are not feeding into machine learning models. 15,348 (44.8%) cylinders have no new shoot, thus labelled with “0”. 2,329 (6.8%) cylinders have one new shoot (labelled “1”). There are fewer cylinder samples, whose new shoot number is larger than “1”: 321 (0.94%) cylinders have 2 new shoots; 54 (0.16%) cylinders have 3 new shoots; 7 (0.02%) cylinders have 4 new shoots; 2 cylinders have 5 new shoots; only 1 cylinder has 6 new shoots on it. Due to the extremely rare samples with a high number of new shoots, we label those cylinders that have more than 4 shoots with new shoot number 4.

Owing to the limited volume of data we acquired, the majority of the items labelled with new shoot numbers from “0” to “4” must feed into machine learning models (16,558 items representing 26 trees). Nevertheless, we reserved 2 trees (1,504 items) as an evaluation dataset. This evaluation dataset was only used to validate the results (see section 3), not to train the model. The dataset for machine learning was further divided into a training set (13,246 items) and a testing set (3,312 items, with a test size of 0.2). The testing set prevented overfitting the models to the given data.

**Figure 4:**
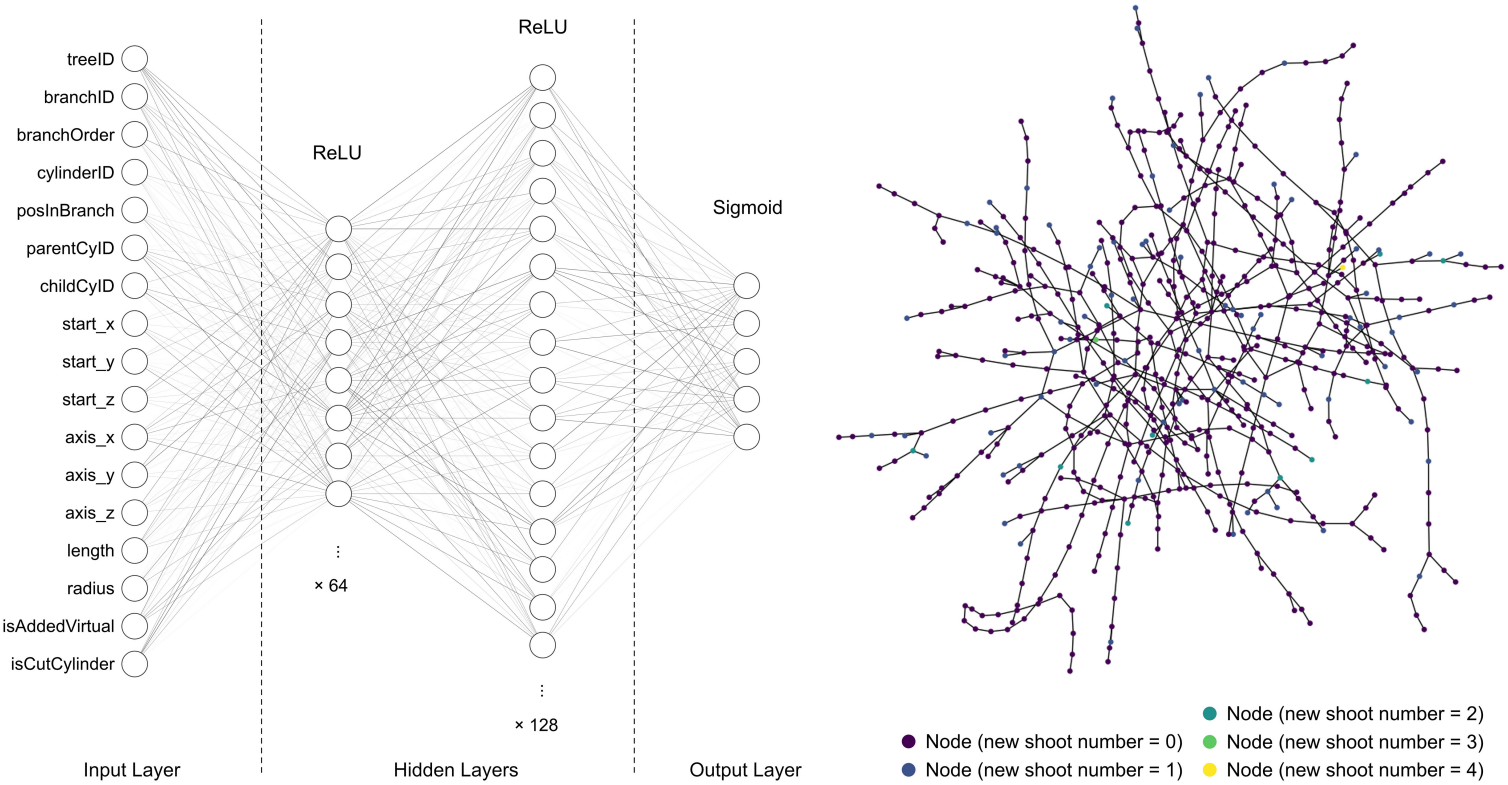
Architecture of the ANN (left) and Graph (right) of one tree used in our test.

For getting a quick overview of the performances across a wide range of classification models in machine learning on the dataset, we used lazy predict (Pandala, 2023) to run scikit-learn (Pedregosa et al., 2011) to compare 25 common classification models with their default settings, including GaussianNB, NearestCentroid and LGBMClassifier. Besides, we tested a basic Artificial Neural Network (ANN) model built with Keras (Chollet, 2015). It consisted of two hidden layers with 64 and 128 nodes, respectively (see **Figure 4** left). In addition, to examine a graph neural network (GNN) model, the dataset for each tree was processed to a graph (Salama, 2021), where every cylinder item was a node connected to its parent and children (the node connection for one tree is illustrated in **Figure 4** right). These graph data were fed into a GNN model named “baseline classifier” (see **Supplementary Table 2.1**), including 39,512 trainable and 1,174 non-trainable params.

We tested all these classification models in two manners of labelling: binary labels that only classify cylinders if they will or will not grow new shoots; multiclass labels that categorize cylinders based on the exact number of new shoots ranging between 0 to 4.

## 3 Results

**Figure 5:**
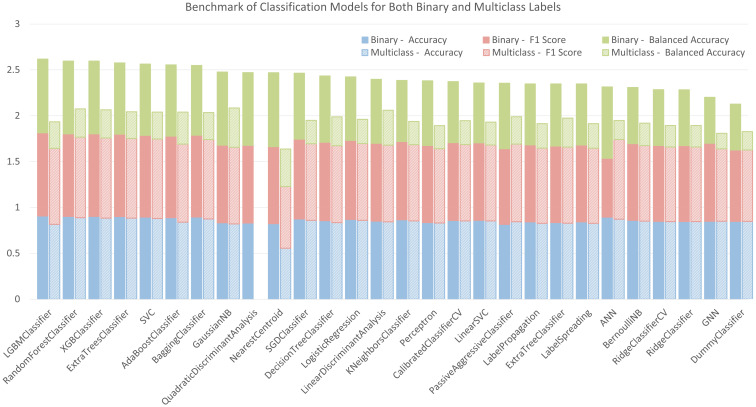
Benchmark of tested classification models for binary and multiclass labelling.

The accuracy, balanced accuracy, and F1 Score (weighted average F1 score for multiclass labels) of the tested models in a default setting or with a basic architecture (see section 2.4) are listed in **Figure 5**. Each scoring index ranges between 0 and 1. 1 is the best score, meaning that all the shoot labels are correctly predicted. On the contrary, 0 is the worst score, representing no correct prediction. In the figure, these models are shown in descending order from the left to the right according to their total scores in classifying binary labels. Among the three sub-scores, accuracy reflects an overall rate of true predictions for all labels. Our datasets are imbalanced in terms of different label numbers. Therefore, balanced accuracy, which gives equal weights to the true prediction rates for each label, is also an important indicator in evaluating their performances. The F1 score is another effective index for the imbalanced classifications but attaches more importance to true positives (predicting the cylinders with new shoots correctly), while it ignores the true negatives (predicting the cylinders with zero shoot correctly). Based on these benchmark scores, LGBMClassifier and GaussianNB have top scores for predictions with binary and multiclass labels, respectively. The confusion matrix of the LGBMClassifier with binary labels in the testing set is shown in **Supplementary Table 3.1**. The confusion matrix of the GaussianNB model with multiclass labels in the testing set is shown in **Supplementary Table 3.2**.

To validate these two models further, we applied the trained LGBMClassifier model and GaussianNB model to the evaluation set with binary and multiclass labels respectively. The results of the evaluation are visually illustrated in **Supplementary Figure 3**. Their performance metrics including precision, recall, and F1 Score for every label on the validation set are shown in **Supplementary Tables 3.3** and **3.4**. The accuracy, balanced accuracy, and weighted F1 score of both models with the evaluation set (only 2 trees) have a maximum of around 10% difference from the scores on the benchmark.

## 4 Discussion

To be able to meaningfully interpret and evaluate the results, it is first necessary to discuss the specific conditions of the dataset and resulting limitations.

The following factors may impact the accuracy of the extracted geometrical data from the trees: 1) To prevent browsing the tree barks, protecting covers were installed below 2 meters around the tree trunks. This might have caused the diameter measured at trunk cylinders to be slightly overestimated. However, we assume that this has no influence on the prediction model. 2) Minor swinging of the branches by wind during the LiDAR scanning might have caused outliers or might have led to overestimating the diameter of the smaller branches. Although the point clouds were denoised through SOR filters, this does not guarantee the full deletion of these outliers and could then cause inexistent branches in the cylindrical models. 3) Aligning the same trees with different geometries in the two years has been a nonstandard manual process so far, which can cause inconsistency in change detection and identification of parent cylinders. A possible alternative to detect these changes is comparing the occupancy grids (Hirt et al., 2021).

The total number of cylinders for training the models was limited to 16,558, representing 26 trees. The percentage of the negative label “0” makes up more than 92% of the total items, causing an unbalanced rate for the number of positive samples (less than 2500 items). Unfortunately, these are all available data from the nursery.

Most importantly, the collected dataset in two consecutive years reflects the growth of these trees under almost identical environmental conditions and pruning regimes. More specifically, the temperature, water content in the soil, wind direction and speed as well as the time of pruning are all the same for these trees. This means that our method can predict the resprouting pattern of this kind of table-topped plane trees grown under the same conditions as in this study. In case of any changes in the factors mentioned above, it is unclear so far how accurate the prediction will be. For instance, the model may not predict the growth of the same trees in the following year. Horticultural experience even shows that a change in the time of pruning of only one or two weeks can have a significant impact on the growth of new shoots, especially if there is also a change in weather conditions (e.g., heat or drought immediately after pruning).To understand whether those environmental factors could also be addressed in a prediction model in the same approach, these environmental data must be collected and coupled with a larger quantity of trees. This hints at an upcoming step in this study.

Except for the barriers in data quality, its available amount, and environmental descriptors above, the following technical difficulties in this computational workflow may be worth paying attention to. 1) Merging the QSMs of one tree scanned at different times is not robust. To improve this, a reference-based cylinder fitting function should be considered. In this way, the later QSM of the tree can be built based on its previous QSMs. Then, the girth growth for each cylinder can be precisely linked from time to time following this idea. 2) For pruning and resprouting issues, positions and lengths of actual internodes are more helpful than current cylinders that contain only geometrical information and lack connections to physiological processes. Axillary buds can possibly be identified in detailed, colorful images of the tree trunks or branches. These can be used for fitting cylinders faithful between physiological nodes of the plants. 3) The LGBMClassifier and GaussianNB models are lightweight and efficient. They have shown the best performance on our relatively small dataset. If they were applied to bigger databases, their accuracy remains to be evaluated, especially in handling a higher diversity in tree ages and shapes. 4) After predicting the position of new shoots, our current model did not answer the ongoing growth of those shoots. It is possible to combine a L-system growth simulation (Boudon et al., 2012) with the QSM (see Shu et al., 2022). In this way, our model can be integrated as a tool to interrupt a natural growth through branch pruning.

Finally, our current model is only the first step in understanding resprouting patterns after one specific artificial disturbance, namely pruning of table-topped trees. Nonetheless, we are optimistic that the approach has great potential for further development and application (see e.g. Yazdi et al., 2023). The application of such a model is not limited to repeating what the gardeners can already do but goes beyond knowledge boundaries regarding the resprouting strategy of trees after disturbances. This can hopefully be achieved through gathering massive tree database (e.g. Yazdi et al., 2024). By searching this database, the “digital gardener” will likely find evidence to support its predictions in a more complex context. In agricultural automation, robots are already self-navigating through an orchard (Ye et al., 2023) and picking fruits (Meng et al., 2023; Wang et al., 2023). Following this trend, this study may offer hints about how pruning decisions could be made by the “digital gardener”. For this far vision, an open-source and uniform data platform about trees [e.g., tree information modeling (Shu et al., 2022)] is required.

## 5 Conclusion

Resprouting patterns are vital in understanding the regeneration of trees after natural and artificial disturbances. The interrelationships are very complex, involving the primary status of hormones, the redistribution of resources, and timing issues. Until now, no single model has addressed all these factors with a physiological approach. However, for centuries, gardeners and practitioners have been trained to prune trees based on their intuitive predictions. They are able to do so based on accumulated knowledge working with trees. In this study, we gave it a first try addressing whether computational models, especially machine learning models, could gain similar knowledge as practitioners from horticulture: what are the location and numbers of new shoots after pruning? Which model would achieve the best performance?

For this purpose, we scanned a group of annually pruned plane trees at a tree nursery with LiDAR. The detailed geometry and topology of the branches were extracted through quantitative tree models. The trimmed branches and new shoots were detected through comparison between the scans in two consecutive years. This information was finally labelled on a dataset for training multiple classification models.

We tested 25 common classification models in machine learning with default settings. Additionally, 1 ANN model and 1 GNN model with the most basic architectures were also tested. Among these models, except for two, all other models have an accuracy and an F1 score higher than 80%. For balanced accuracy, the average score of all the models was ca. 70% for binary labels; for multiclass labels, the average was 28.3%.

From the results, we can conclude that for the collected dataset, most models work well in telling the position of new shoots but are not accurate in describing the actual shoot numbers at the specific location. For the best scored models with binary labelling, the LGBMClassifier can predict the position of new shoots with an accuracy of 90.8% and a balanced accuracy of 80.3%. For predicting the exact number of the shoots, the GaussianNB Model performs the best. The accuracy is 82.1% because most cylinders should have the shoot number 0. However, the balanced accuracy is reduced to 42.9%.

The innovation of this work was to identify the tree cases in a controlled environment for studying their quantitative reactions to disturbances. It is the first study to address the resprouting pattern prediction with QSM data. To achieve this, it is highly novel to combine QSMs of different times of a tree into one. It is also of significant value to indicate a primary comparison of the performances of various machine learning models in this task.

The applicability of the current model is limited to the studied site, environmental conditions, tree species and form, and the pruning time. In the next step, a larger amount of tree data is being collected in the city of Munich to analyse how this approach can be extended to a broader scope, maybe addressing some of the environmental factors. In a further vision, a massive database of the “digital gardener” would push forward the boundaries of knowledge in understanding the resprouting strategies of trees facing natural and artificial disturbances.

## Data availability statement

The original contributions presented in the study are included in the article/**Supplementary Material**. Further inquiries can be directed to the corresponding author.

## Author contributions

QS: Conceptualization, Data curation, Investigation, Methodology, Project administration, Resources, Software, Validation, Visualization, Writing – original draft, Writing – review & editing. HY: Data curation, Investigation, Software, Validation, Writing – review & editing. TR: Funding acquisition, Resources, Supervision, Writing – review & editing. FL: Conceptualization, Funding acquisition, Project administration, Resources, Supervision, Writing – review & editing.
