# Supplementary material for: Predicting resprouting of Platanus × hispanica following branch pruning by means of machine learning
Source: Front Plant Sci. 2024 Mar 7;15:1297390. doi: 10.3389/fpls.2024.1297390 (PMC10954810; doi:10.3389/fpls.2024.1297390)
Supplement: Supplementary file 1 [file DataSheet_1.zip › Supplementary Figure 1.pdf]

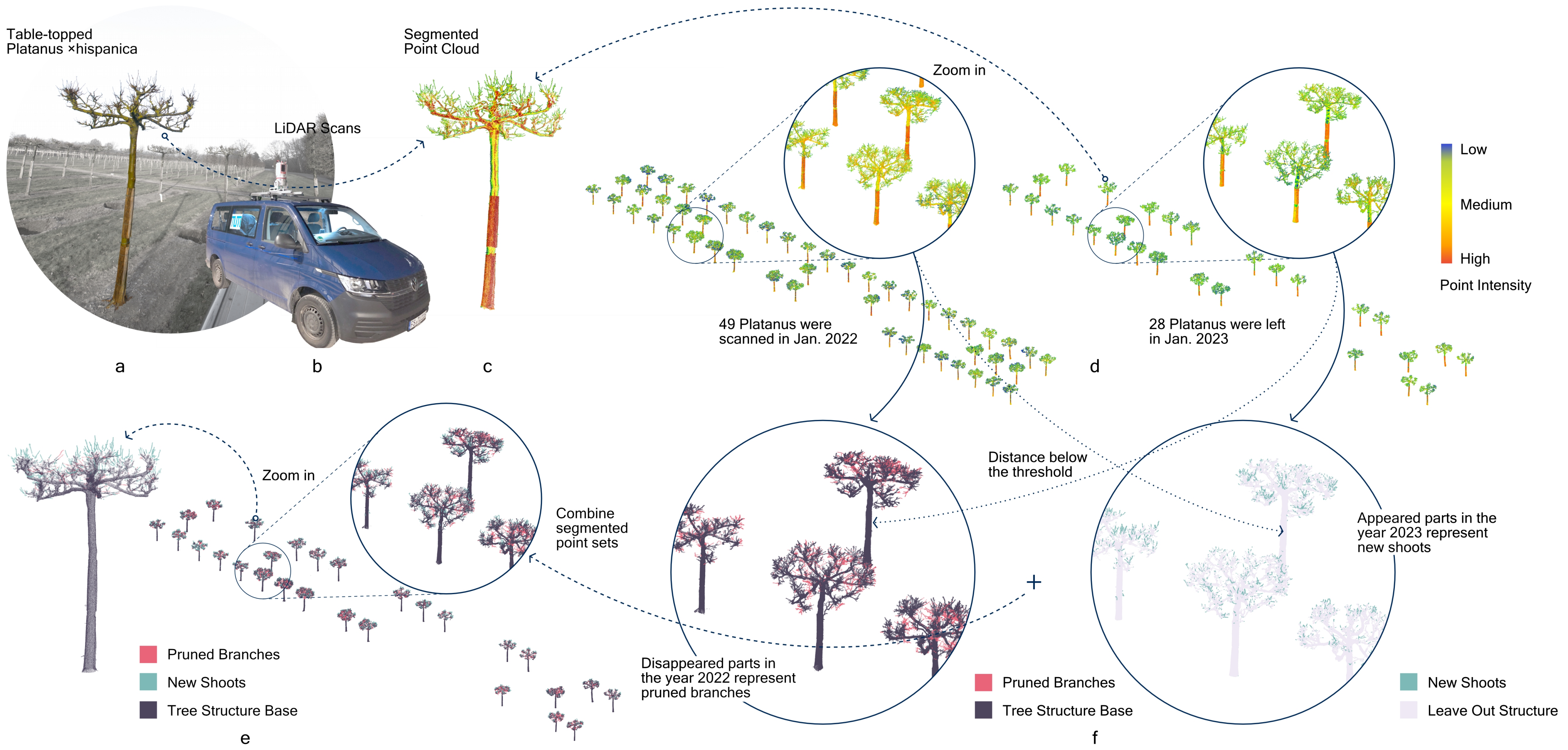

Table-topped
Platanus ×hispanica
LiDAR Scans
Segmented
Point Cloud
Zoom in
Low
Medium
High
Point Intensity
49 Platanus were
scanned in Jan. 2022
28 Platanus were left
in Jan. 2023
a
b
c
d
Zoom in
Combine
segmented
point sets
Distance below
the threshold
Appeared parts in the
year 2023 represent
new shoots
+
Disappeared parts in
the year 2022 represent
pruned branches
Pruned Branches
New Shoots
Tree Structure Base
Pruned Branches
Tree Structure Base
New Shoots
Leave Out Structure
e
f
