# Supplementary material for: Predicting resprouting of Platanus × hispanica following branch pruning by means of machine learning
Source: Front Plant Sci. 2024 Mar 7;15:1297390. doi: 10.3389/fpls.2024.1297390 (PMC10954810; doi:10.3389/fpls.2024.1297390)
Supplement: Supplementary file 1 [file DataSheet_1.zip › Supplementary materials.DOCX]

Supplementary Material

# Supplementary Data

The raw data of the studied tree cases in Quantitative Tree Models is presented in the csv file named “AdaptQSM_Table topped platanus x hispanica.csv”.

# Supplementary Figures and Tables

## Supplementary Tables

Table 2.1. Architecture of the GNN model in our test.

| Layer (type) | Output Shape | Param | Connected to |
| --- | --- | --- | --- |
| input_features (InputLayer) | [(None, 11)] | 0 | [] |
| ffn_block1 (Sequential) | (None, 64) | 5228 | ['input_features[0][0]'] |
| ffn_block2 (Sequential) | (None, 64) | 8832 | ['ffn_block1[0][0]'] |
| skip_connection2 (Add) | (None, 64) | 0 | ['ffn_block1[0][0]', 'ffn_block2[0][0]'] |
| ffn_block3 (Sequential) | (None, 64) | 8832 | ['skip_connection2[0][0]'] |
| skip_connection3 (Add) | (None, 64) | 0 | ['skip_connection2[0][0]', 'ffn_block3[0][0]'] |
| ffn_block4 (Sequential) | (None, 64) | 8832 | ['skip_connection3[0][0]'] |
| skip_connection4 (Add) | (None, 64) | 0 | ['skip_connection3[0][0]', 'ffn_block4[0][0]'] |
| ffn_block5 (Sequential) | (None, 64) | 8832 | ['skip_connection4[0][0]'] |
| skip_connection5 (Add) | (None, 64) | 0 | ['skip_connection4[0][0]', 'ffn_block5[0][0]'] |
| logits (Dense) | (None, 2) | 130 | ['skip_connection5[0][0]'] |

Table 3.1. Confusion Matrix of LGBMClassifier with Binary Labels in Testing Set

|  | Negative / 0 | Positive / 1 |
| --- | --- | --- |
| Negative / 0 | 2883 (79.8%) | 170 (4.7%) |
| Positive / 1 | 209 (5.8%) | 351 (9.7%) |

Table 3.2. Confusion Matrix of GaussianNB Model with Multiclass Labels in Testing Set

|  | 0 | 1 | 2 | 3 | 4 |
| --- | --- | --- | --- | --- | --- |
| 0 | 2458 (74.2%) | 309 (9.3%) | 33 (1.0%) | 0 (0%) | 1 (0%) |
| 1 | 153 (4.6%) | 258 (7.8%) | 24 (0.7%) | 0 (0%) | 3 (0.1%) |
| 2 | 29 (0.9%) | 27 (0.8%) | 5 (0.2%) | 0 (0%) | 0 (0%) |
| 3 | 4 (0.1%) | 4 (0.1%) | 0 (0%) | 0 (0%) | 1 (0%) |
| 4 | 2 (0.1%) | 1 (0%) | 0 (0%) | 0 (0%) | 0 (0%) |

Table 3.3. Performance Metrics of LGBMClassifier on Binary Labels in Evaluation Set

| label | precision | recall | f1-score | support |
| --- | --- | --- | --- | --- |
| 0 | 0.95 | 0.94 | 0.95 | 1276 |
| 1 | 0.68 | 0.75 | 0.71 | 228 |
| accuracy |  |  | 0.91 | 1504 |
| macro average | 0.82 | 0.84 | 0.83 | 1504 |
| weighted average | 0.91 | 0.91 | 0.91 | 1504 |

Table 3.4. Performance Metrics of GaussianNB Classifier on Multiclass Labels in Evaluation Set

| label | precision | recall | f1-score | support |
| --- | --- | --- | --- | --- |
| 0 | 0.94 | 0.88 | 0.91 | 1276 |
| 1 | 0.42 | 0.60 | 0.50 | 199 |
| 2 | 0.05 | 0.04 | 0.04 | 24 |
| 3 | 0.00 | 0.00 | 0.00 | 4 |
| 4 | 0.00 | 0.00 | 0.00 | 1 |
| accuracy |  |  | 0.82 | 1504 |
| macro average | 0.28 | 0.30 | 0.29 | 1504 |
| weighted average | 0.85 | 0.82 | 0.83 | 1504 |

## Supplementary Figures

**Supplementary Figure 1.** An overview of the workflow for point cloud acquiring and pre-processing.

**Supplementary Figure 2.** Correlations between each two attributes of the dataset. The number of new shoots is indicated with the colour, a darker colour represents a larger number of new shoots on the cylinder. The value “-1” is marked as pruned out cylinders.

**Supplementary Figure 3.** Visualizing the evaluation of the LGBMClassifier and GaussianNB models in predicting resprouting patterns. The ground truths are illustrated in the middle. The binary predictions using LGBMClassifier are drawn on the lower left side while the multiclass predictions using GaussianNB model are drawn on the upper right.
