# Supplementary figures and images for: Predicting resprouting of Platanus × hispanica following branch pruning by means of machine learning
Source: Front Plant Sci. 2024 Mar 7;15:1297390. doi: 10.3389/fpls.2024.1297390 (PMC10954810; doi:10.3389/fpls.2024.1297390)

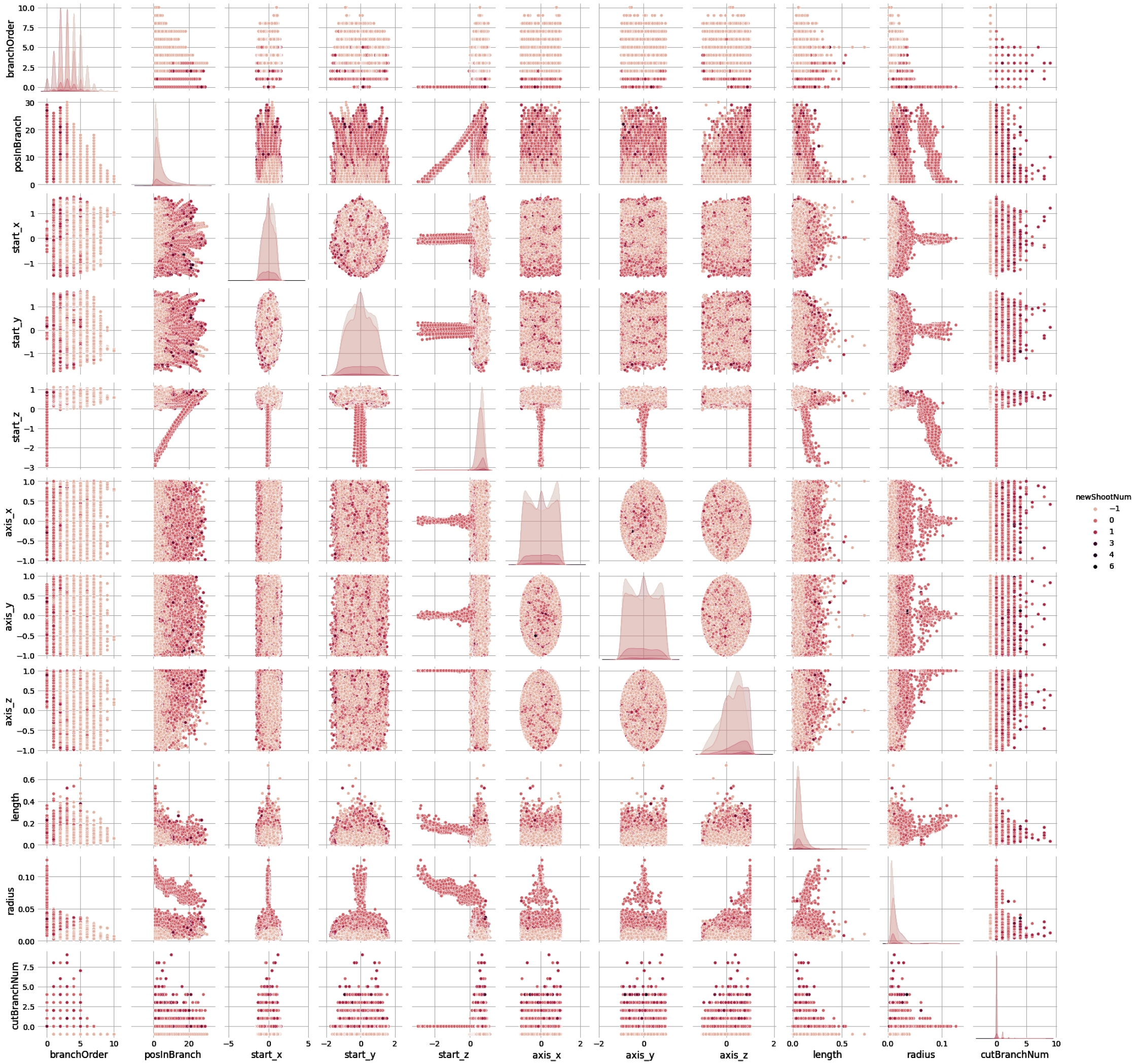

Supplement: Supplementary file 1 [file DataSheet_1.zip › Supplementary Figure 2.pdf]

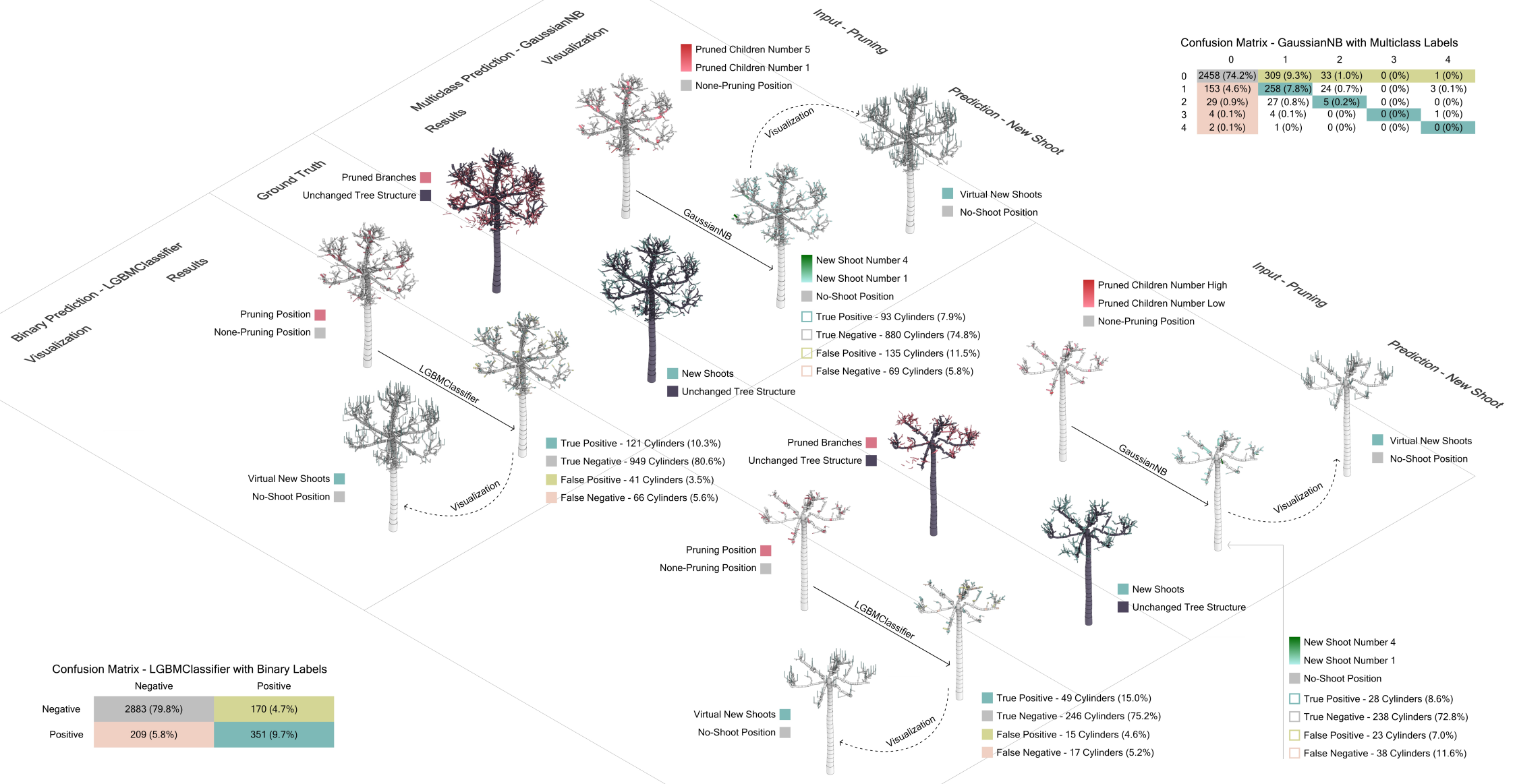

Supplement: Supplementary file 1 [file DataSheet_1.zip › Supplementary Figure 3.pdf]
